# An RNA-informed dosage sensitivity map reflects the intrinsic functional nature of genes

**DOI:** 10.1016/j.ajhg.2023.08.002

**Published:** 2023-08-23

**Authors:** Danyue Dong, Haoyu Shen, Zhenguo Wang, Jiaqi Liu, Zhe Li, Xin Li

**Affiliations:** 1 CAS Key Laboratory of Computational Biology, Shanghai Institute of Nutrition and Health, University of Chinese Academy of Sciences, Chinese Academy of Sciences, 320 Yue Yang Road, Shanghai, 200031, China

**Keywords:** dosage sensitivity, homeostasis, gene function

## Abstract

Understanding dosage sensitivity or why Mendelian diseases have dominant vs. recessive modes of inheritance is crucial for uncovering the etiology of human disease. Previous knowledge of dosage sensitivity is mainly based on observations of rare loss-of-function mutations or copy number changes, which are underpowered due to ultra rareness of such variants. Thus, the functional underpinnings of dosage constraint remain elusive. In this study, we aim to systematically quantify dosage perturbations from *cis*-regulatory variants in the general population to yield a tissue-specific dosage constraint map of genes and further explore their underlying functional logic. We reveal an inherent divergence of dosage constraints in genes by functional categories with signaling genes (transcription factors, protein kinases, ion channels, and cellular machinery) being dosage sensitive, while effector genes (transporters, metabolic enzymes, cytokines, and receptors) are generally dosage resilient. Instead of being a metric of functional dispensability, we show that dosage constraint reflects underlying homeostatic constraints arising from negative feedback. Finally, we employ machine learning to integrate DNA and RNA metrics to generate a comprehensive, tissue-specific map of dosage sensitivity (MoDs) for autosomal genes.

## Graphical abstract

**Figure:**
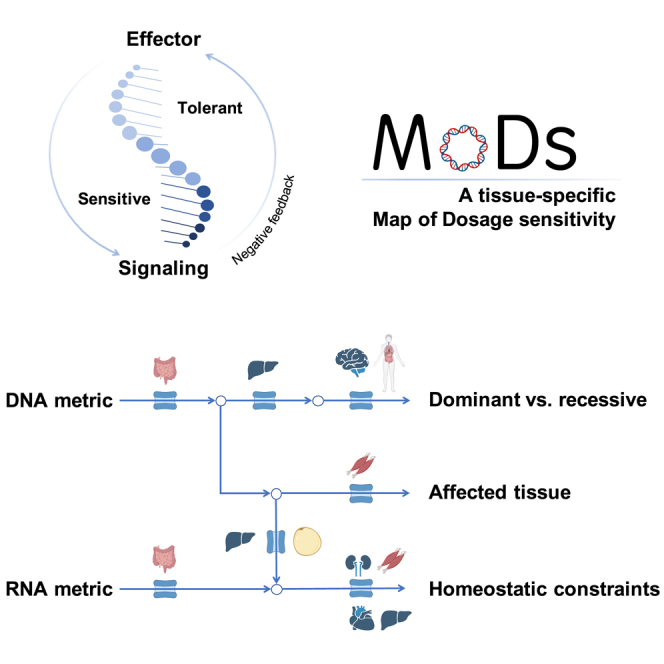

## Teaser

We introduce a tissue-specific map of dosage sensitivity (MoDs) of genes. MoDs can be used to better understand pathogenic modes of inheritance (dominance vs. recessiveness), to infer which tissues may be affected and to inform models of the underlying homeostatic mechanism.

## Introduction

What gives rise to dominance and recessiveness is a long-standing question in genetics.^1^ Currently, the largest number of reported genetic disorders observed in human populations are attributed to loss-of-function (LoF) mutations causing alterations in gene dosage.^2^^,^^3^^,^^4^ Meanwhile, the majority of common disease loci detected by genome-wide association studies (GWASs) are found in the noncoding region of the genome, presumably also affecting disease susceptibility by altering gene dosage.^5^^,^^6^ Understanding dosage constraints and the impact of dosage alterations is therefore crucial for uncovering the genetic basis of human diseases.

Measurement of dosage constraint was previously mainly based on the presence of LoF mutations^7^^,^^8^ and copy number variations (CNVs)^9^^,^^10^ in human populations. However, those mutations are rare and such studies would still be underpowered even after sequencing the entire human population.^4^

By contrast, *cis*-acting regulatory variants (expression quantitative trait loci or *cis*-eQTL) cause changes in gene expression typically by altering regulatory elements such as enhancers and promoters^11^^,^^12^^,^^13^^,^^14^ and are also very informative for understanding underlying dosage constraints. While LoF variants can be considered as global (cross-tissue) knockouts at the DNA level, *cis*-eQTLs can be seen as conditional knockouts or tissue-specific perturbations, which inform tissue-specific dosage constraints and in addition are more prevalent than rare LoF mutations or CNVs.^15^^,^^16^

In this study, we utilize *cis*-regulatory variants to assess tissue-specific gene dosage constraints and present an RNA-informed gene dosage sensitivity map across human tissues, which more directly reflects dosage constraints of genes under physiological conditions. We further explored functional implications of dosage constraints and how negative feedback and homeostatic constraints influence dosage sensitivity. Finally, we integrated both DNA and RNA metrics into a comprehensive reference map of tissue-specific dosage constraints (MoDs) for autosomal genes. The tissue-specific dosage sensitivity map can be used to better understand pathogenic modes of inheritance (dominance vs. recessiveness), to infer which tissues may be affected, and to inform models of the underlying homeostatic mechanism.

## Material and methods

### *cis*-eQTL effect size calculation

The RNA metric for dosage sensitivity is based on eQTL effect sizes, with larger effect sizes indicating more dosage tolerance. We measured eQTL effect sizes in 49 tissues from GTEx v.8 data release.^14^ To account for varying sample sizes across tissues, we used the full set of multi-tissue QTL recalibration by METASOFT^17^ which incorporates cross-tissue correlation and maximizes detection power. For each tissue, we measured the effect size at eQTLs with METASOFT m-value > 0.9.

Biologically interpretable effect sizes for eQTLs are defined as the log allelic fold change (aFC^18^), the ratio between the expression of the haplotype carrying the alternative allele to the one carrying the reference allele in log2 scale. aFC was calculated for all eQTLs per gene per tissue using both total expression (eQTL) and allele-specific expression (ASE) methods.

### ASE-based calculation

GTEx v.8 haplotype phasing data were used to determine the allelic expression for each eQTL allele by adding the allelic expression from all phased heterozygous SNPs within the gene, with a pseudo-count of 1 added to both the reference and alternative eQTL haplotype counts. aFC is calculated by two formulations: the ratio of the allelic expression summation in all individuals or the median ratio across all individuals:

$$aFC=\frac{\sum_{i=1\ldots \;N}C_{1,i}}{\sum_{i=1\ldots \;N}C_{0,i}}$$

$$aFC=\underset{i=1\ldots N}{median}\frac{C_{1,i}}{C_{0,i}}$$

*C*_0,*i*_ and *C*_1,*i*_ represent the allelic expression of the haplotypes carrying the reference or alternative alleles of an eQTL for individual *i* in N individuals, where i∈{1,2,…,N}. Confidence intervals were computed as regular binomial proportion confidence intervals for the first formulation and as Wald intervals for the second formulation.

### Gene-expression-based calculation

We used gene expression data, in the form of transcript per million (TPM), as quantified by GTEx v.8 consortium pipeline. The effect size was computed by linear regression, adjusted for the potential confounding effect of hidden PEER factors,^19^ the top three principal components of the genotype matrix, the sex of the donor, the sequencing platform used for WGS, and the WGS library construction protocol.

$$Y_{i}=b_{0}+b_{1}t_{i}+\sum_{m=1}^{M}\alpha_{i,m}P_{m}+\sum_{k=1}^{3}\beta_{i,k}G_{k}+\gamma_{i}Sex+\delta_{i}Platform+\mu_{i}Libray+\varepsilon_{i}$$

*Y*_*i*_ is the expression for individual *i* in N individuals. *t*_*i*_ is the number of alternative alleles in each individual: $t_{i}\in \left\{0,1,2\right\}$. $P_{m}$ is the m^th^ PEER factor, *G*_*k*_ is the top k genotype principal components.

Allelic fold change (aFC) was calculated from the slope and the intercept (together with their confidence interval) as fitted by the linear regression.

$$aFC=\frac{2b_{1}}{b_{0}}+1$$

We additionally calculated an aFC estimate under a nonlinear model by using the aFC.py tool.^18^

To ensure high confidence estimates, at each eQTL we intersected the confidence intervals of all methods to derive the most likely range of the aFC estimate, abs(log2(aFC)) ∈ [*L*_*i*_*, U*_*i*_] for an eQTL variant *i*. To obtain the maximum eQTL effect at each gene, we selected the eQTL variant *i* of the maximum estimation lower bound *L*_*i*_.

### Expression outliers and rare variants effects

Expression outliers were identified following the same data normalization and batch effect correction pipeline as in our previous work.^12^^,^^13^ Within each tissue, we transformed the expression values by taking the log base 2 of the TPM values plus 2 (log2(TPM + 2)). We selected only autosomal lincRNA and protein-coding genes and included only those with at least 6 reads and a TPM greater than 0.1 in at least 20% of individuals. We scaled the expression of each gene to have a mean of 0 and a standard deviation of 1. As outliers are prone to technical bias and batch effects, to account for both known and hidden covariates, we used a linear model to remove the effects of PEER factors, the top three genotype principal components, sex, and the genotype of the strongest *cis*-eQTL per gene in each tissue, obtaining expression residuals. Finally, we re-scaled the expression residuals for each gene to obtain corrected expression Z-scores for each individual per gene per tissue. For each gene, we extracted maximum and minimum Z-scores across individuals per tissue. We identified outliers using the Z-scores for each individual in each tissue. For cross-tissue outliers, we set a threshold of abs(median(Z-scores)) > 2. The effect size of an identified outlier (aFC, expression compared to population mean) was re-calculated on the natural scale of the original TPM to yield a biologically interpretable value.

We restricted our rare variant analyses to individuals of European descent, as they constituted the largest homogeneous population within GTEx dataset. We retained all SNVs and indels that successfully passed quality control in the GTEx VCF (v.8 version). Structural variants were identified in the subset of individuals available from v.7.^20^ Rare variants were defined as having MAF ≤ 0.01 in GTEx, and for SNVs and indels we also required MAF ≤ 0.01 within gnomAD v.3 samples. All rare SNVs and indels were annotated using Ensembl’s Variant Effect Predictor (VEP) v.101^21^ with the loss-of-function transcript effect estimator (LOFTEE) plugin.^15^ Noncoding variants were additionally annotated with CADD^22^ score and conservation scores (Gerp,^23^ PhyloP,^24^ PhastCons^24^).

When evaluating the enrichment of rare variants by category, we applied a 10 kb ± window centered around the gene body. In cases where multiple rare variants were present in proximity to a gene, we categorized each gene-individual pair into a single variant category. Our ordering for assigning categories was as follows: duplications (DUP), copy number variations (CNV), deletions (DEL), breakend (BND, complex rearrangements which cannot be readily classified into those canonical forms of SV), inversions (INV), transposable elements (TE), splice, frameshift, stop, transcription start site (TSS), conserved non-coding (CADD>20 and non-coding), coding, or other non-coding.

### Gene functional classification

Functional categorization of genes was mainly based on the KEGG database.^25^ We classified genes in 14 categories: mitochondrial DNA (mtDNA) genes, transporter, cytokine and hormone, ion-channel, receptor, intracellular signaling (protein kinase and G protein), metabolic enzyme, cytoskeleton, membrane trafficking, extracellular matrix, exosome, mitochondrial-related genes, DNA replication repair-related genes, and RNA transcription- and translation-related genes. Considering that some genes may fall into multiple groups, we set an order of priority to functional categories and assigned a unique category to each gene. Functional categories are specified in Table S1.

lincRNAs were annotated according to a previous study,^26^ which manually combined a set of 954 genes from lncRNAdb,^27^ the HUGO gene nomenclature committee, and recent work that identified functional lncRNA genes through CRISPR screens, plus 5 genes found in the literature that were not covered in these three sources.

Inheritance mode for mendelian diseases and their associated phenotypes are compiled from OMIM and HPO databases^2^^,^^28^ (Table S1).

The list of genes in the energy homeostasis system is based on KEGG pathways and manual curation, as summarized in Table S2. Effector genes were from the pathways of glycolysis, gluconeogenesis, glycogenolysis, gluconeogenesis, fatty acid biosynthesis, and lipolysis. Signaling genes were from pathways of insulin/glucagon signaling, regulation of lipolysis in adipocytes, adipocytokine signaling, PPAR signaling, AMPK signaling, and epinephrine signaling.

### UK Biobank association analysis

UK Biobank^29^ (UKB) data were obtained under application number 54622. Informed consent was obtained as part of the enrollment process for the UK Biobank.

We used variant calls for 200,643 subjects who had undergone exome sequencing and genotyping by the UK Biobank Exome Sequencing Consortium.^30^ The UK Biobank cohort was filtered to only unrelated individuals (based on field 22020) with self-reported white British ancestry (field 22006), resulting in a total of 138,032 samples.

Glycated hemoglobin (HbA1c; field: 30750) and glucose (field: 30740) were taken as the maximum observed across visits. The criteria for diabetes included the presence of ICD10 codes E10–E14 (field 41202), recorded use of diabetes medication (field 6177, 6153), diabetes diagnosed by a doctor (field 2443), nurse interview codes indicating diabetes (field 1220: any diabetes, 1222: T1D, 1223: T2D), or HbA1c ≥ 48 mmol/mol.

For the LoF analysis, all variants were annotated using VEP v.101^21^ with LOFTEE.^15^ To ensure high confidence that variants truly resulted in a loss-of-function (LoF), we considered only ultra-rare LoF variants (MAF ≤ 0.0001 in UKB) and we further filtered out variants located in exons with low expression (pext^31^ value smaller than 0.1) or in the last exon of a gene. Association of LoF variants with a phenotype was evaluated by linear regression considering sex and age as covariates.

For the GWAS analysis, summary statistics were obtained from the Neale Lab server available at http://www.nealelab.is/uk-biobank. We re-calculated effect sizes on the natural scale of the phenotype to obtain a biologically interpretable measure.

### Machine learning approaches to infer tissue-specific dosage tolerance

We compiled a list of features containing tissue-specific gene-level attributes for utilization in the machine learning model to predict tissue-specific dosage constraint. These features are divided into six main categories: genomic, expression, chromatin, protein, mutational constraint, and function (Table S3). Gene expression features were derived from GTEx v.8. Median expression and median absolute deviation were used to measure expression levels and variability. All chromatin features were based on the Roadmap Epigenomics Project^32^ using the core 15-state ChromHMM^33^ model, where 27 Roadmap tissues were mapped to 26 GTEx tissues. Other epigenomic features were extracted from a previous study.^10^

We trained and applied a LightGBM model^34^ (Figure S10) to predict tissue-specific dosage tolerance based on gene-level features. The positive training set consisted of high-confidence dosage-tolerant genes in tissues, meeting either of the following criteria: (1) genes that have large effect eQTLs (abs(log2(aFC)) >1) in a tissue or (2) genes tolerant to LoF variation (gnomAD LoF observed/expected lower bound > 0.5). Genes that do not meet these criteria were considered as potentially dosage constrained and formed the negative set. As negative instances in the training data could also contain tolerant genes due to sparseness of dosage changing variants, the model is expected to be conservative in calling genes that are truly tolerant. We performed random down sampling on the negative set to balance the training data. LightGBM was implemented using a Python package.^34^ 5-fold cross-validation based on RandomizedSearchCV^35^ was performed to select the optimal hyperparameters. We held out 30% of the data to evaluate model performance (Figure S10). Importance of features is measured by SHAP^34^ values.

### An RNA-integrated dosage constraint map

Naturally existing variants of any type are not saturated by random mutation. To fully capture multiple lines of evidence for dosage tolerance, we integrated into a final assessment all the information from DNA/RNA metrics and model inference, namely tolerance to loss of function (gnomAD o/e LoF lower bound^15^) and effect size of eQTL (machine-learning-inferred tissue-specific tolerance based on multi-omics features).

We scaled each metric using a rank normalization approach, where we ranked the genes based on each metric and then normalized the ranks by rank(x)/N, where N is the total number of observations. For each gene, the maximum rank-scaled tolerance score of three metrics was reported as a final tolerance score. We eventually produced tolerance scores for a total of 16,448 autosomal protein-coding genes across 49 tissues. Genes that do not have a gnomAD LoF evaluation, were not expressed, or were without eQTL in any GTEx tissues were not scored.

## Results

### Protein-truncating and regulatory variants reflect concordant underlying dosage constraint

**Figure 1:**
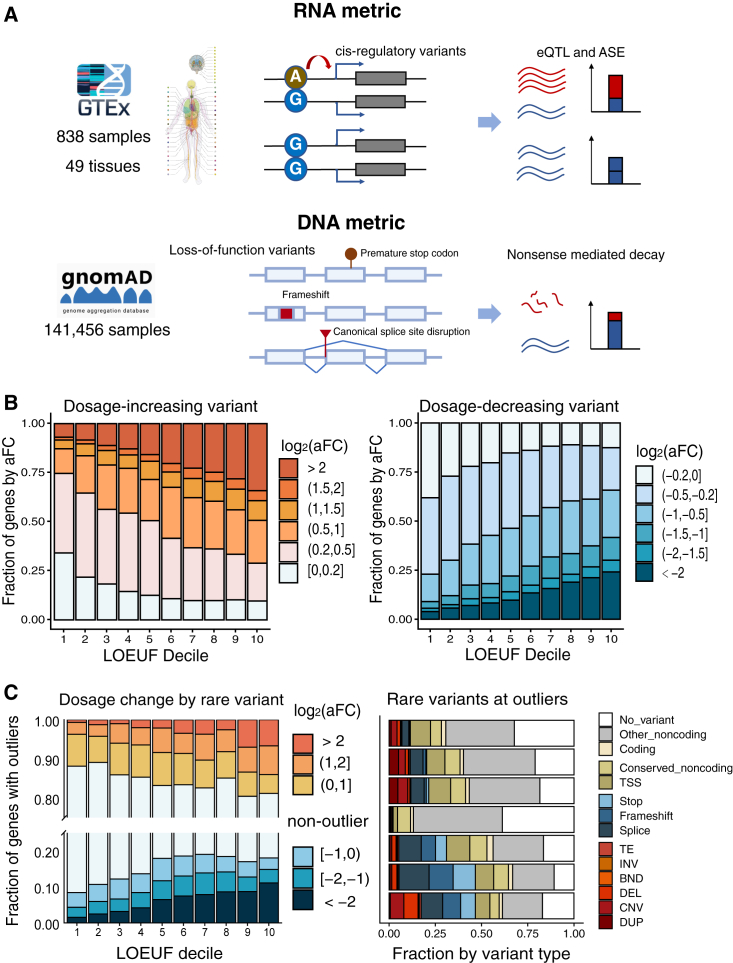
DNA vs. RNA metrics for measuring dosage constraints (A) Schematic illustration of RNA metrics (*cis*-regulatory variants) and DNA metrics (LoF variants) for measuring dosage constraints. (B) DNA vs. RNA metrics at 16,448 autosomal protein-coding genes. RNA metrics (aFC, allelic fold change induced by *cis*-regulatory variants) are calculated in tissues from GTEx; larger aFC indicates more dosage tolerant. For the RNA metric, the aFC in each direction (dosage increase or decrease, direction is with respect to reference allele of a *cis*-regulatory variant) is shown in left and right panels. For each gene, we take the maximum aFC (by effect size |aFC|) across tissues (median aFC across tissues are shown in Figure S2). The DNA metric is the degree of depletion of LoF variants as measured by observed/expected upper bound fraction (LOEUF) from gnomAD. Smaller LOEUF indicates higher dosage constraint. (C) Dosage changes driven by rare variants. Left panel shows effect sizes (mean aFC over tissues) of genes at under-expression and over-expression outliers. Right panel shows underlying rare variants at those expression outliers. TSS, transcription start site; TE, transposable element; BND, breakend; DEL, deletion; CNV, copy-number variation; DUP, duplication; INV, inversion.

We systematically measured mRNA dosage perturbations by *cis*-regulatory variants (Figure 1A). As naturally existing *cis*-regulatory variants are depleted of detrimental effects as a result of purifying selection, their effect sizes can indicate a tissue-specific range of dosage tolerance. To quantify genetically induced dosage changes, we computed allelic fold change (aFC)^18^ arising from *cis*-regulatory variants (eQTLs) in the general population using transcriptome sequencing data of 49 tissues of 838 individuals from GTEx project.^14^ We derived aFC from both total expression level (eQTL) and allele-specific expression (ASE) to mitigate the potential power loss of each approach (see material and methods). The eQTL approach requires sufficient allele frequency of a *cis*-regulatory variant while the ASE approach requires sufficient RNA read depth at exonic sites to achieve a confident measure. We overlapped 95% confidence intervals of two measures to obtain a most likely range [*L*_*i*_, *U*_*i*_] of the effect size estimate for a *cis*-regulatory variant *i*. To obtain the dosage-tolerance range of a gene, we took the maximum aFC of all *cis*-regulatory variants of a gene, but each at its confidence interval lower bound *L*_*i*_ to account for estimation uncertainty.

We compared dosage constraints by the DNA metric and RNA metrics in each tissue. DNA-based constraints are derived from the degree of depletion of LoF variants among 141,456 exomes as generated by gnomAD project.^15^ Our rationale was that LoF variants (by introducing premature stop codons, frameshifts, canonical splice site disruptions, and other protein-truncating effects) are global knockouts which measure the lowest dosage-tolerance range across tissues (in other words, LoF is intolerated as long as dosage is constrained in any individual tissue), while the RNA metric indicates tolerance range in individual tissues. RNA and DNA metrics of dosage constraints show highly concordant patterns (Figure 1B), with large effect RNA perturbations (large aFC eQTLs) increasingly depleted at genes where LoF variants are not tolerated. At each gene, the maximum aFC (in each direction) of all tissues is shown in Figure 1B (median aFC across tissues is shown in Figure S2), for 16,448 autosomal protein-coding genes binned by both their DNA (LOEUF) and RNA (aFC) metrics. Across tissues, we observe consistent patterns of reduced eQTL effect sizes at LoF-intolerant genes (Figures 1B and S1; Table S4), suggesting that RNA and DNA metrics are concordant measures of underlying dosage constraints. Of note, this depletion of large effect eQTLs is not because of reduced statistical power due to low gene expression levels,^14^^,^^36^ as LoF-intolerant genes are actually cross-tissue more highly expressed than other genes.^15^ As intolerance to LoF (haploinsufficiency) only reflects sensitivity to dosage decreases, but the RNA metric (eQTLs) can measure dosage sensitivity in both directions, we observed that LoF-intolerant genes are depleted of both dosage-decreasing and dosage-increasing effects, indicating that dosage is usually concurrently constrained or relaxed in both directions.

eQTLs only capture effects at common variants. To reveal dosage-changing effects of rare variants which can potentially have larger effects,^12^^,^^13^ we examined the most extreme individuals (highest or lowest expression outliers) at each gene among 838 individuals in the GTEx cohort (see material and methods). Major driving variants underlying those outliers are duplications and promoter variants for over-expression outliers, versus deletion, stop codon, splice site, and frameshift variants for under-expression outliers (Figure 1C). Either large effect over-expression or under-expression outliers induced by rare variants also show a concordant trend of depletion at LoF-intolerant genes, suggesting a consistent underlying dosage constraint.

Together, this implies that dosage constraint is an intrinsic property of a gene, regardless of being measured from protein-truncating variants (LoF) or regulatory variants. However, the RNA metric does provide tissue-specific information beyond that of DNA metric, which will be further discussed in the next section. Thus, transcriptome-based measures can substantially complement LoF-based DNA metrics and provide a tissue-specific evaluation of dosage constraint.

### Functional role is a major determinant of dosage constraint

**Figure 2:**
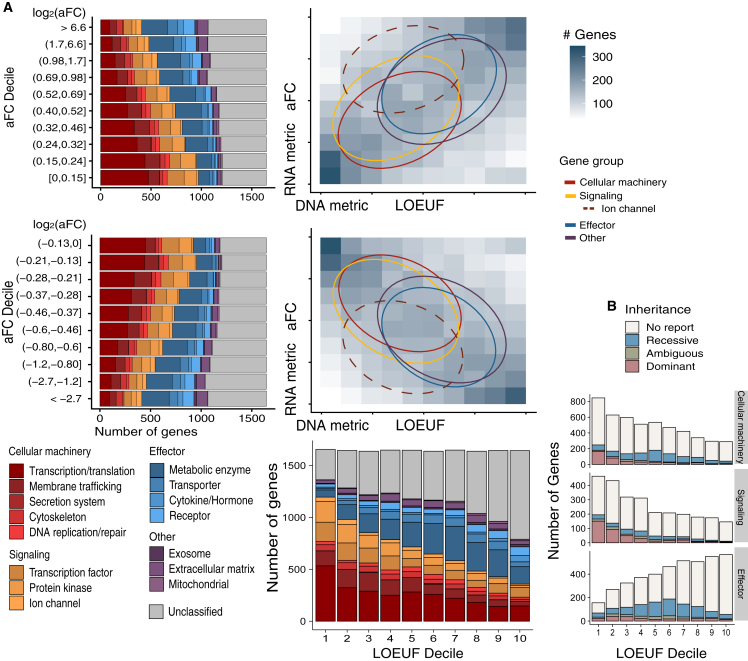
Relationship of dosage constraint with gene function (A) Dosage constraints of 16,448 autosomal protein-coding genes of different functions on DNA and RNA metrics. We show both directions of dosage change as measured by the RNA metric. As the RNA metric is the maximum aFC across tissues (median aFC shown in Figure S3), it can reveal possible tissue-specific tolerance beyond the DNA constraint metric. Circles mark 50% of the genes around center of mass of a functional category. (B) Number of genes containing variants associated with known dominant or recessive Mendelian diseases by functional category: cellular machinery, signaling, and effector genes.

To further explore the functional logic underlying dosage constraints, we mapped 16,448 autosomal protein-coding genes onto RNA and DNA dosage constraint spectrums. We observe a notable divergence of dosage constraints between two groups of genes, with cellular machinery, transcription factors, protein kinases, and ion channels (signaling genes) being dosage sensitive while metabolic enzymes, transporters, cytokines, and receptors (effector genes) being significantly more tolerant to dosage perturbations (Figure 2A). The measured dosage sensitivity is consistent with observed Mendelian diseases in human populations, with the majority of variants in transporters, metabolic enzymes, cytokines, and receptors being associated with recessive diseases, while dominant disorders are more frequently reported for variants in genes found among the cellular machinery, transcription factors, protein kinases, and ion channels groups (Figure 2B). To exclude the possibility that such a difference is due to potential decoupling of DNA/mRNA to protein abundance by post-transcriptional regulation, we compared RNA to protein correlation in GTEx proteomics data^37^ between different functional categories. Similar RNA to protein correlation (>0.5) was observed across all functional groups (Figure S4), indicating that both the RNA and DNA metrics can well reflect final protein dosage constraints. As we have previously observed, dosage sensitivity usually occurs concurrently in both directions of change, which is also true in the context of different functional groups. Effector genes can tolerate both dosage increase or decrease, while signaling genes are intolerant to either direction of change (Figure 2A).

**Figure 3:**
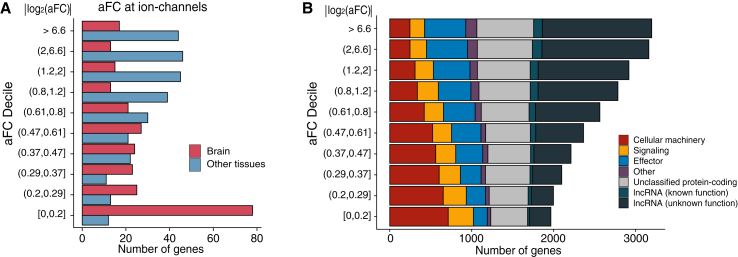
The RNA metric provides tissue-specific information on dosage constraint for protein-coding and non-protein-coding genes (A) Dosage constraints of ion channels in brain and non-brain tissues (maximum aFC among each tissue group). (B) Comparison of dosage constraints of lncRNA with protein-coding genes. For each gene, we show maximum aFC across tissues. Median aFC across tissues is shown in Figure S5.

As LoF variants are DNA (cross-tissue) knockouts, tolerance of LoFs indicates tolerance across all tissues while intolerance of LoF variants within a gene can arise from dosage constraint at any individual tissue. As a result, the DNA (LoF)-based metric reflects the most stringent constraint across tissues, but it does not specifically identify the constrained tissue. The RNA metric can reveal where exactly dosage is constrained. Ion channels are dosage change intolerant as indicated by the DNA metric (intolerant to LoF), but the RNA metric further elucidates that a large portion of ion channels are constrained specifically in brain, but not constrained (having large eQTLs) in other tissues (Figure 3A, p < 1e−15). We further examined dosage constraints at long noncoding RNAs (lncRNAs),^26^ which cannot be measured by LoF-based DNA metrics. lncRNAs are in general much less constrained than protein-coding genes (Figure 3B, p < 1e−15). lncRNAs of known functions are slightly more constrained than other lncRNAs (p = 0.012). Together, these observations suggest that dosage constraints are widely divergent and are largely determined by the functional nature of genes.

### Dosage constraint informs underlying homeostatic constraint

We observe that metabolic enzymes, transporters, cytokines and hormones, and receptors are generally more tolerant of genetic dosage perturbations; however, they are final effectors to execute strict homeostatic constraints. We therefore further explored how those effector genes can tolerate dosage perturbations and whether this could instead be a functional requirement by using energy homeostasis as a model.

We compiled a thorough list of experimentally established metabolic enzymes and transporters of the energy homeostasis system (glycolysis, glycogenesis, glycogenolysis, gluconeogenesis, lipolysis, and fatty acid biosynthesis) and their negative feedback axes (insulin/glucagon, PPAR, AMPK, adipocytokine, and epinephrine signaling) from the KEGG database^25^ and by manual curation (Figure 5A; Table S2).

**Figure 4:**
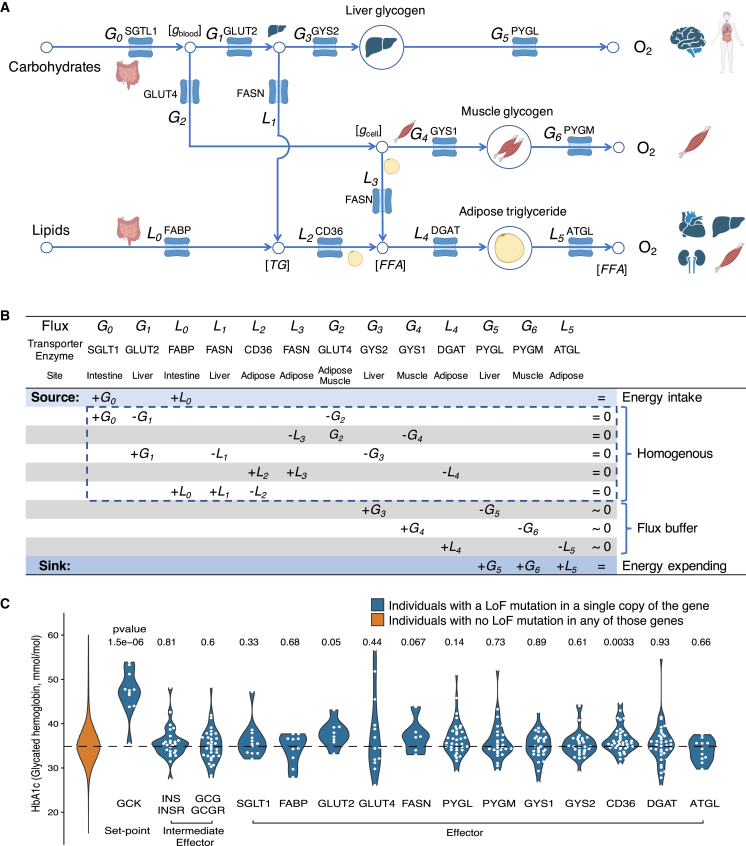
Dosage constraints in energy homeostasis system (A) Energy flux balance constraints. Each arrow represents an energy flux, which is marked as *L*_*i*_ for lipids and *G*_*j*_ for glucose. Effector genes encoding metabolic enzymes or transporters marked on each arrow (flux) control that flux. Chemical concentrations at each node are marked as [g_blood_], blood glucose; [g_cell_], intracellular glucose; [FFA], free fatty acids; [TG], triglyceride. A detailed description of their physiology is provided in Figure S6. (B) Equation system and solution space of energy flux under the law of conservation of energy. (C) Effect of single-copy LoF mutations within effector genes and set-point genes. Glycated hemoglobin (HbA1c, baseline level ∼35 mmol/mol) measures of individuals in UK Biobank with single functional copies (due to LoF mutations) of effectors, intermediate effectors, and set-point genes. HbA1c reflects long-term blood glucose levels; other glycemic measures exhibit similar patterns (as shown in Figure S7). Association p value of LoF variants with a phenotype was evaluated by linear regression considering sex and age as covariates.

The homeostasis system of energy flux (*L*_*i*_ for lipids and *G*_*j*_ for glucose, as defined in Figure 4A), essential effectors (metabolic enzymes and transporters, functional details described in Figure S6) controlling the flux, and the corresponding equation system are specified in Figures 4A and 4B (Figure S6). The solution space (under the law of conservation of energy) is maintained by negative feedback at the homogeneous part (constant = 0) of the equation system, which confines 8 variables (energy input *L*_*0*_*, G*_*0*_ being arbitrary) in a solution space of 3 degrees of freedom. For the homogeneous part (energy turnover), in brief, glucose absorbed by the intestine *G*_*0*_ is partially taken up by the liver *G*_*3*_ and muscle *G*_*4*_ as glycogen and partially converted by the liver *L*_*1*_ and adipose tissue *L*_*3*_ to lipids, such that *G*_*0*_ = *G*_*3*_ + *L*_*1*_ + *L*_*3*_ + *G*_*4*_; absorbed lipids by the intestine *L*_*0*_ together with *L*_*1*_ + *L*_*3*_ is taken up by adipose tissue such that *L*_*0*_ + *L*_*1*_ + *L*_*3*_ = *L*_*4*_.

Homeostasis is a basic requirement to sustain all forms of life. In the example of energy homeostasis, the biological control system to balance the flux (to reach equilibrium, i.e., ∑*in-flux* = ∑*out-flux* for all nodes in Figure 4A) follows the general rules of a flow network,^38^^,^^39^^,^^40^ with the solution space (constraints) defined by an equation system as specified in Figure 4B. Flux is controlled by effectors (metabolic enzymes or transporters, as marked in Figure 4A) and homeostasis is maintained by adjusting those effectors through a negative feedback mechanism. Negative feedback toward a set point is a general strategy to solve an equation system (as in Figure 4B), where the set point is a reference value for a control system (e.g., blood glucose concentration = 4.5 mmol/L, body temperature = 37°C, diastolic blood pressure = 80 mmHg). Substrate concentration accumulating (at a node in Figure 4A) above the set point indicates ∑*in-flux* > ∑*out-flux* while depleting below a set point indicates ∑*in-flux* < ∑*out-flux*, so the relationship above or below a set point informs the control system of the right direction of adjustment for effectors controlling in- or out-flux such that a new solution can be reached (all constraints are satisfied at the homogeneous part of the equation system in Figure 4B, i.e., ∑*in-flux* = ∑*out-flux* for all nodes in Figure 4A, a solution space of 8 flux variables with 3 degrees of freedom). Homeostasis being the basis of life and the eventual goal of a control system, failure to reach the solution space is a disease status, for example, *G*_*0*_ > *G*_*3*_ + *L*_*1*_ + *L*_*3*_ + *G*_*4*_ is diabetes, *L*_*0*_ + *L*_*1*_ + *L*_*3*_ > *L*_*4*_ is fatty liver disease or hyperlipidemia. Regulation of flux through effectors (metabolic enzymes or transporters) is physically achieved through allosteric/covalent changes (*K*_*cat*_, effector efficiency) or *trans*-locational/transcriptional changes ([*E*], effector abundance) following Michaelis-Menten kinetics *flux* $=\frac{\left[S\right]}{K_{m}+\left[S\right]}K_{cat}\left[E\right]$.

**Figure 5:**
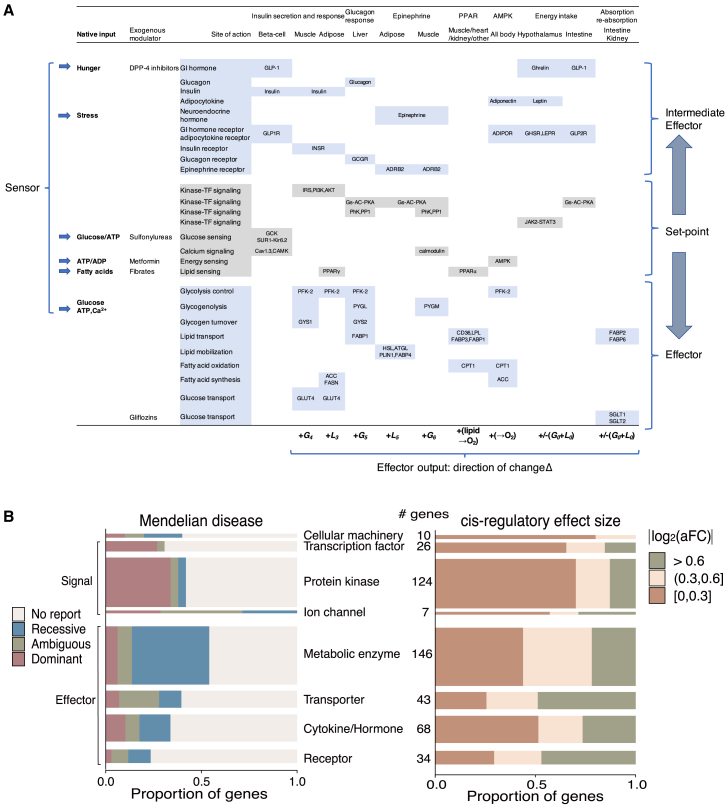
Dosage constraint and negative feedback (A) Major negative feedback axes of the energy homeostasis system. A negative feedback axis (columns) consists of the effector part (intermediate effector) and the signaling part (set point). Effector and signaling parts are color shaded as blue and gray, respectively. (B) Dosage constraint at known genes of the energy homeostasis system. Genes are divided into the dosage-sensitive group (cellular machinery, protein kinases, ion channels, and transcription factors) and the resilient group (metabolic enzymes, transporters, cytokines, and receptors). Bin size is scaled to the number of genes in each functional category. Left panel shows the proportion of genes associated with Mendelian diseases by mode of inheritance and functional category. Right panel shows eQTL effect sizes (aFC) of genes in each functional category (maximum aFC among major organs of energy homeostasis of intestine, stomach, liver, pancreas, muscle, adipose tissues, and hypothalamus).

As blood glucose is a commonly measured set point, we first tested whether the solution space (homeostatic constraints) could be interrupted by a single copy loss (from LoF mutations) within genes encoding components of negative feedbacks. We classify genes into effectors (transporters and metabolic enzymes), intermediate effectors (cytokines and receptors), and set point determinants based on their roles in negative feedbacks (Figure 5A). Examining individuals with a single copy of a LoF mutation in a gene of interest among the natural population of 138,032 participants from UK Biobank (see material and methods), we can observe that single copy losses of effectors or intermediate effectors do not interrupt glucose homeostasis (Figure 4C, blood glucose, i.e., substrate concentration at node [*g*_blood_] in Figure 4A still preserved at set point, implying satisfied flux constraint *G*_*0*_ = *G*_*1*_ + *G*_*2*_ = *G*_*3*_ + *L*_*1*_ + *L*_*3*_ + *G*_*4*_). Interruption of the set point determinants (GCK, beta-cell glucose sensor) of the negative feedback, however, impairs balance of the system resulting in abnormal accumulation of glucose concentration (p < 1e−5, Figures 4C and S7) in blood (node [*g*_blood_] in Figure 4A, suggesting unbalanced flux *G*_*0*_ > *G*_*1*_ + *G*_*2*_ beyond set point). Tolerance of single copy LoF mutations within effector genes (transporters and metabolic enzymes) along energy flux routes suggests that the solution space is not only robust to environmental perturbations (arbitrary energy intake of *L*_*0*_*, G*_*0*_) but also robust to genetic perturbations, as long as the negative feedback (Figure 5A) is preserved, which maintains the solution space (solves the equation system) by requiring the direction of flux adjustment by effectors to be opposite to the difference of a chemical potential to the set point. In the example of the insulin axis, the negative feedback is requiring: sign([blood glucose] – set point) = – sign(Δ*G*_*3*_(GYS2_liver_) + Δ*G*_*4*_(GLUT4_muscle_GYS1_muscle_) + Δ*L*_*1*_(FASN_liver_) + Δ*L*_*3*_(GLUT4_adipose_FASN_adipose_)), physically achieved through allosteric, covalent, *trans*-locational, or transcriptional changes to the effectors (directly or through intermediate effectors, Figures 4A and 5A).

Examining all negative feedback axes (458 genes, Figure 5A; Table S2) of the energy homeostasis system, we can observe that a majority of reported Mendelian diseases caused by mutations within effector genes are recessive while those caused by mutations in signaling (set point) genes are dominant (Figure 5B left; Figure S8A), concordant with a general divergent pattern of dosage sensitivity between effector and signaling genes (Figure 2A). Such dosage constraint divergence is also revealed by the RNA metrics (Figure 5B right), at major energy homeostasis organs (intestine, stomach, liver, pancreas, muscle, adipose tissues, and hypothalamus), as eQTL effect sizes at effector genes are also significantly larger. The phenotypic effects of common variants that induce dosage changes (eQTLs) can be further assessed by a GWAS (see material and methods). As expected, most selectively neutral common variants lead to very minor phenotypic effects irrespective of their eQTL effect sizes (Figure S8B), justifying the use of naturally existing common variants for measuring dosage-tolerance range. Dosage resilience of metabolic enzymes and transporters (effectors) or cytokines and receptors (intermediate effectors) reflects existence of negative feedback systems, and on the other hand, the dosage-sensitivity metric can be used to provide information on their roles (signaling vs. effector) in a homeostatic control system.

### A comprehensive RNA-informed tissue-specific dosage-sensitivity map of autosomal genes

Dosage perturbations induced by *cis*-regulatory variants can substantially complement the existing DNA dosage constraint metrics, but the RNA metric still relies on naturally existing genetic variants, which are not saturated by random mutation. Current metrics are therefore an underestimate of tolerance range if solely based on existing observations of dosage-altering variants.

To account for such information sparseness and further refine the metric, we employed a LightGBM-based^34^ machine learning model to incorporate both the DNA and RNA metrics of genes together with their functional embedding. The model was trained on the most confident positive set of dosage-tolerant genes (large aFC in a tissue, or a significant amount of LoF mutations in populations) utilizing functional categories, genomic and tissue-specific transcriptomic features (see material and methods), such that it can effectively infer dosage tolerance where naturally existing variants are sparse (Figure S10). Among those features, gene expression levels in each tissue are important predictors of tissue-specific dosage constraint, with lower expression level suggesting lower constraint.

**Figure 6:**
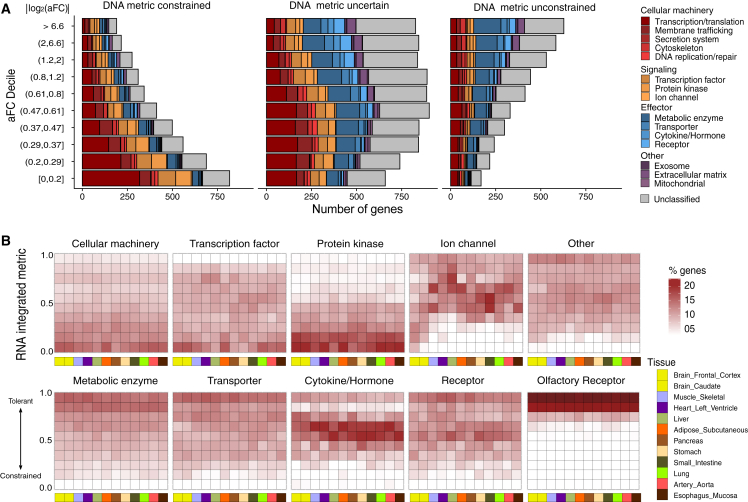
An integrated metric of RNA and DNA dosage constraints (A) The RNA metric complements the DNA metric. Genes on the X axis (DNA metric) are ranked by their depletion of LoF (observed/expected, estimation lower bound). The left bin is solely composed with genes o/e upper bound <0.5 (DNA metric high confidence constrained), while the right bin is solely with those with o/e lower bound >0.5 (DNA metric high confidence unconstrained). The middle bin is composed of genes where the DNA metric is underpowered (o/e lower bound < 0.5 and upper bound > 0.5 or ambiguous confidence interval due to short gene length. The DNA metric confidence interval as affected by gene length is illustrated in Figure S12). The y axis is the RNA metric (aFC). For each gene, maximum aFC across tissues is plotted (median aFC across tissues is shown in Figure S11). (B) Distribution of genes among functional categories in 12 representative tissues as determined by the RNA integrated metric. The RNA integrated metric (MoDs) is produced by a machine learning model, with larger values indicating more dosage tolerant (scaled by rank from 0 to 1).

The RNA metric complements the DNA metric (which are underpowered for short genes such as cytokines or hormones) and more importantly the RNA metric provides a tissue-specific measure of dosage constraint. To illustrate how the RNA metric provides information beyond that provided by the DNA metric, we divided 16,488 autosomal protein-coding genes into three bins (Figure 6A), where the DNA metric can confidently identify genes as constrained (left) or tolerant (right) or is underpowered to make a determination (middle). For those genes constrained on the DNA metric, the RNA metric reveals tissue specificity based on the presence of large-effect eQTLs (e.g., ion channels in non-brain tissues). For those unconstrained genes confident on the DNA metric (global knockout tolerant), the RNA metric will accordingly report those genes as unconstrained. For those genes where the DNA metric is underpowered, the RNA metric can distinguish unconstrained (e.g., cytokines and hormones) from constrained (e.g., cellular machinery) ones based on eQTL effect sizes (Figure 6A). The distributions of RNA-integrated metrics (MoDs, combining RNA and DNA metric by machine learning model) among representative tissues is shown in Figure 6B (a complete dosage constraint map in Table S5), where functional category is a major determinant of dosage constraint.

The integrated score (MoDs) reflects a percentile rank of dosage tolerance across all genes (0 being most constrained and 1 being most tolerant), and we would recommend the use of MoDs <0.3 (or >0.3) as an empirical threshold between dosage-sensitive and dosage-tolerant genes. The tissue-specific score can provide information on the mode of inheritance as well as the likely affected tissue or organ for a dosage-altering mutation. If a gene G is dosage sensitive in any tissue T (MoDs < 0.3 in tissue T), G is likely haploinsufficient and tissue T is likely a pathogenic site affected by a mutation of G. If a gene G is dosage tolerant (MoDs > 0.3) in all tissues, G is likely haplosufficient while the affected tissue of knockout of both copies of the gene can be further assessed by normal gene expression level (typically TPM > 1) of G in different tissues. The dosage sensitivity map can also provide information on the functional roles of genes in maintaining homeostasis and differentiate dosage-sensitive genes (signaling genes: cellular machinery, transcription factors, ion channels, and protein kinases) from dosage-tolerant genes (effector genes: metabolic enzymes, transporters, cytokines, and receptors). From the example of energy homeostasis, we expect that though most effector genes are dosage tolerant (haplosufficient) due to a negative feedback mechanism, they are functionally indispensable (double knockout not tolerated), although a few genes at the very tolerant end (such as olfactory receptors, Figure 6B) may truly be dispensable in humans (double knockout tolerated). Most genes also exhibit similar tolerance patterns in both directions of dosage change (over/under-expression). Tissue-specific values of MoDs, aFC, and TPM are publicly available at https://github.com/xlilab/mods (also provided in Table S5).

## Discussion

Adequate assessment of gene dosage sensitivity is crucial for evaluating the potential impact of genetic variants on gene function and for understanding related diseases. In this study, we comprehensively evaluated gene dosage constraints in different tissues by combining an RNA metric for *cis*-regulatory variants with a DNA-based metric of rare LoF variants or CNVs. The RNA metric effectively complements the DNA metric which relies solely on rare LoF variants and more importantly the RNA metric provides a dosage measure with tissue specificity. In addition to modes of inheritance, the dosage sensitivity map can be used to infer affected tissues and inform on the possible pathogenic mechanism.

We highlighted that gene dosage sensitivity is determined by gene function and especially by their roles (signaling vs. effector) in negative feedback axes. The conventional view suggests that dosage constraint reflects evolutionary conservation, such that dosage-tolerant genes might be less important or even redundant. Here, through a systemic survey of gene function and dosage constraint, we propose that tolerance of dosage perturbation is a functional need in a homeostasis system maintained by negative feedback, such that both dosage-sensitive genes (signaling: cellular machinery, transcription factors, protein kinases, and ion channels) and dosage-tolerant genes (effector: metabolic enzymes, transporters, cytokines, and receptors) are functionally indispensable. Among dosage-constrained genes, transcription factors are well known to provide dosage-dependent cues in developmental morphogenesis. Besides that, set points of negative feedbacks in homeostatic systems are usually genetically predefined (e.g., blood glucose = 4.5 mmol, body temperature = 37°C) most often by transcription factors, protein kinases, and ion channels, which are also dosage sensitive. For energy homeostasis, those set-point determinants are GCK/SUR2-Kir6.2 for glucose, PPAR for lipids, and AMPK for ATP, of which corresponding modulating drugs (sulfonylureas, fibrates, and metformin) have already been discovered (Figure 5A). By understanding tissue-specific gene dosage sensitivity, we can gain deeper insights into the mechanisms of common and rare diseases (stress or failure to reach solution space of homeostasis) and make informed decisions about developing effective therapeutics.

Despite the common assumption that GWAS variants act through dosage change, a very limited number of GWAS signals are so far explained by eQTLs.^14^^,^^41^^,^^42^^,^^43^ Beyond the possibilities of incomplete eQTL discovery in specific cell types or pathogenic contexts, recent studies suggest there may be inherent discovery differences between GWASs and eQTL studies.^44^ As observed in individuals with a single copy of a gene affected by a LoF mutation, most dosage-tolerant genes, especially those effector genes in a resilient solution space maintained by negative feedback could be robust to genetic perturbations, and thus be difficult to discover in genetic association studies despite having large effects on gene expression. On the other hand, for dosage-constrained signaling genes, relying on selectively neutral common variants may limit both GWASs and eQTL discovery studies to finding variants with very small effect sizes. The interplay of dosage constraint and selective pressure on common variants diminishes discovery power of eQTL studies and GWASs, highlighting the importance of discoveries from rare diseases and experimental models, especially for homeostatic systems maintained by strong negative feedback.

Finally, we generated a comprehensive, RNA-informed tissue-specific dosage sensitivity measure for autosomal genes, which can serve as a valuable reference of broad utility to human disease research. However, the current dosage sensitivity map is based on bulk tissues under homeostatic conditions from healthy adults in the general population. It will be essential to further extend such a measure to cell-type-specific resolution over developmental or aging processes and other non-homeostatic dimensions.

## Data Availability

An RNA informed map of dosage sensitivity (MoDs) is publicly available at https://github.com/xlilab/MoDs (also provided in Table S5). GTEx (v.8) RNA-seq and WGS data are available from dbGaP (dbGaP: phs000424.v8.p2). GTEx (v.8) eQTL summary statistics were obtained from the GTEx Portal available at https://gtexportal.org/home/datasets. UKB GWAS summary statistics were obtained from the Neale Lab server available at http://www.nealelab.is/uk-biobank.

## Supplemental information

Data S1. Tables S1–S5

Document S2. Article plus supplemental information
