# Supplementary material for: An RNA-informed dosage sensitivity map reflects the intrinsic functional nature of genes
Source: Am J Hum Genet. 2023 Aug 23;110(9):1509–21. doi: 10.1016/j.ajhg.2023.08.002 (PMC10502852; doi:10.1016/j.ajhg.2023.08.002)
Supplement: Document S1. Figures S1–S12 [file mmc1.pdf]

The American Journal of Human Genetics, Volume *110*

Supplemental information

An RNA-informed dosage sensitivity map reflects the intrinsic functional nature of genes

Danyue Dong, Haoyu Shen, Zhenguo Wang, Jiaqi Liu, Zhe Li, and Xin Li

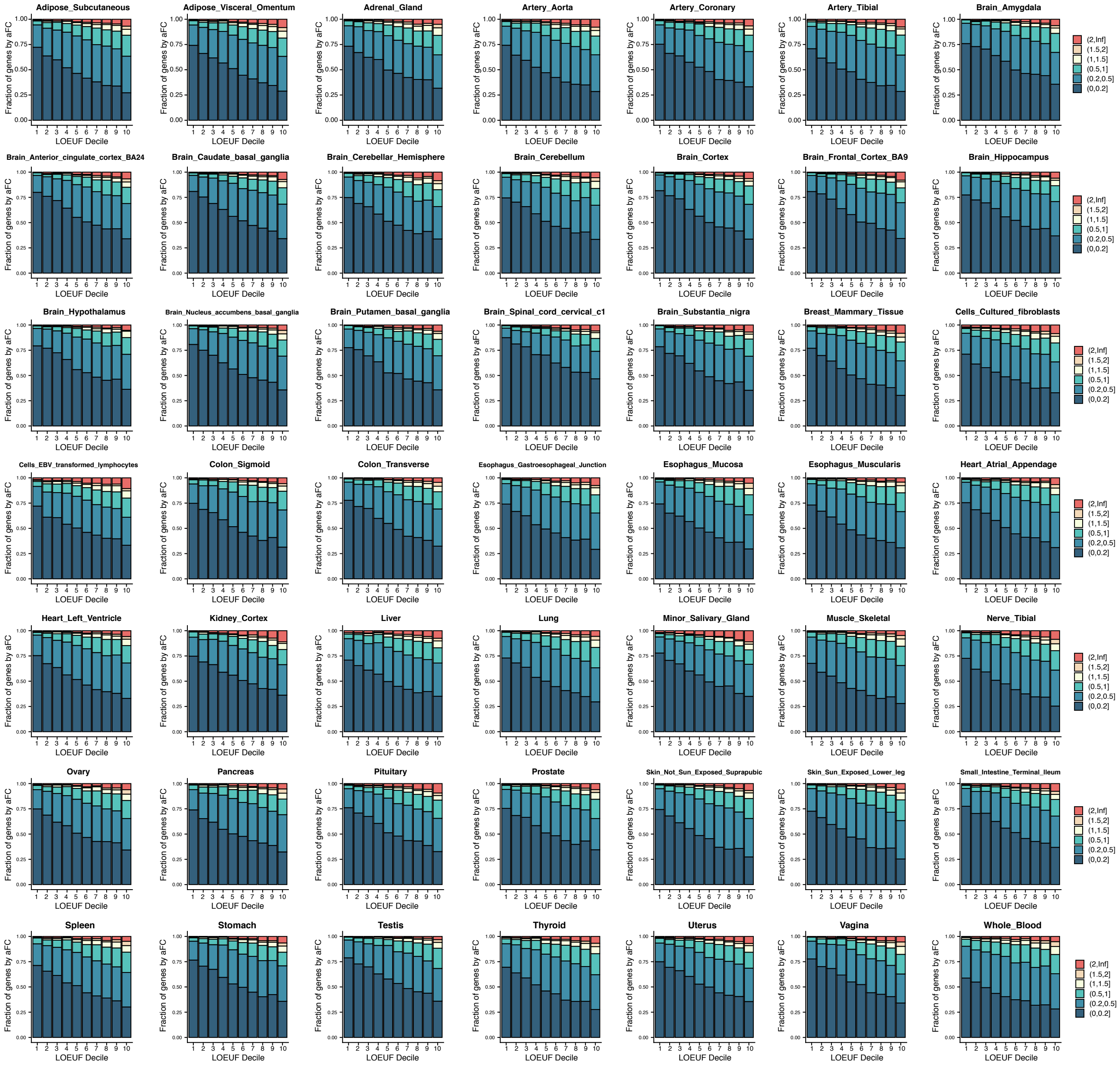

Figure S1. Comparison of DNA and RNA constraint metrics across all tissues. The RNA metric of maximum effect sizes (|log2(aFC)|) of eQTLs at each gene were measured across all GTEx tissues. The DNA metric was observed/expected upper bound (LOEUF) from gnomAD, which indicates degree of LoF tolerance.

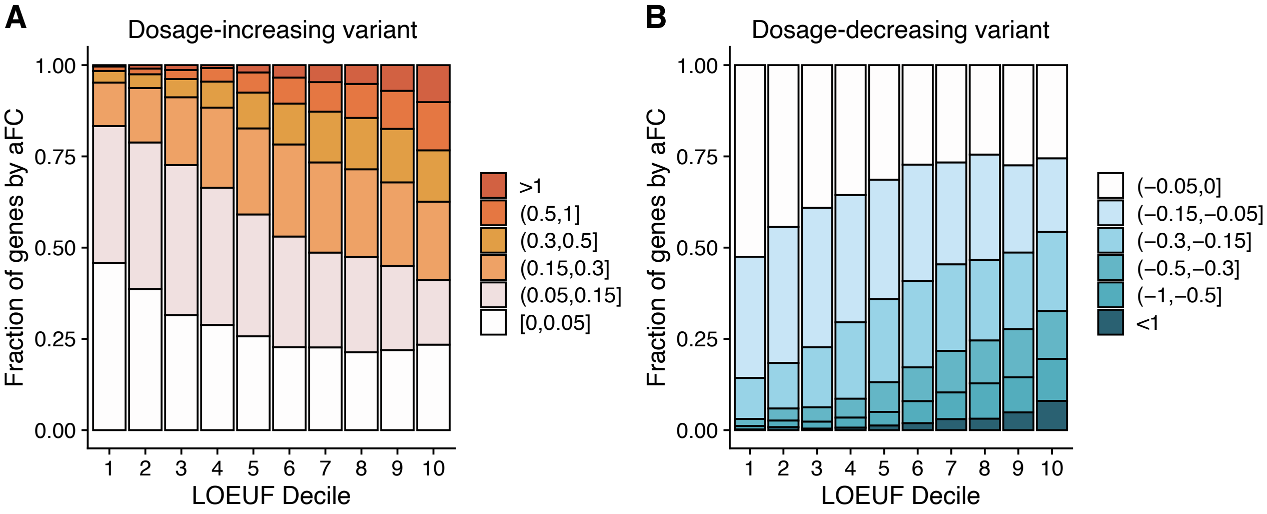

Figure S2. DNA vs. RNA metrics at 16,448 autosomal protein-coding genes. The RNA metric is median aFC across tissues. The DNA metric is observed/expected upper bound fraction (LOEUF).

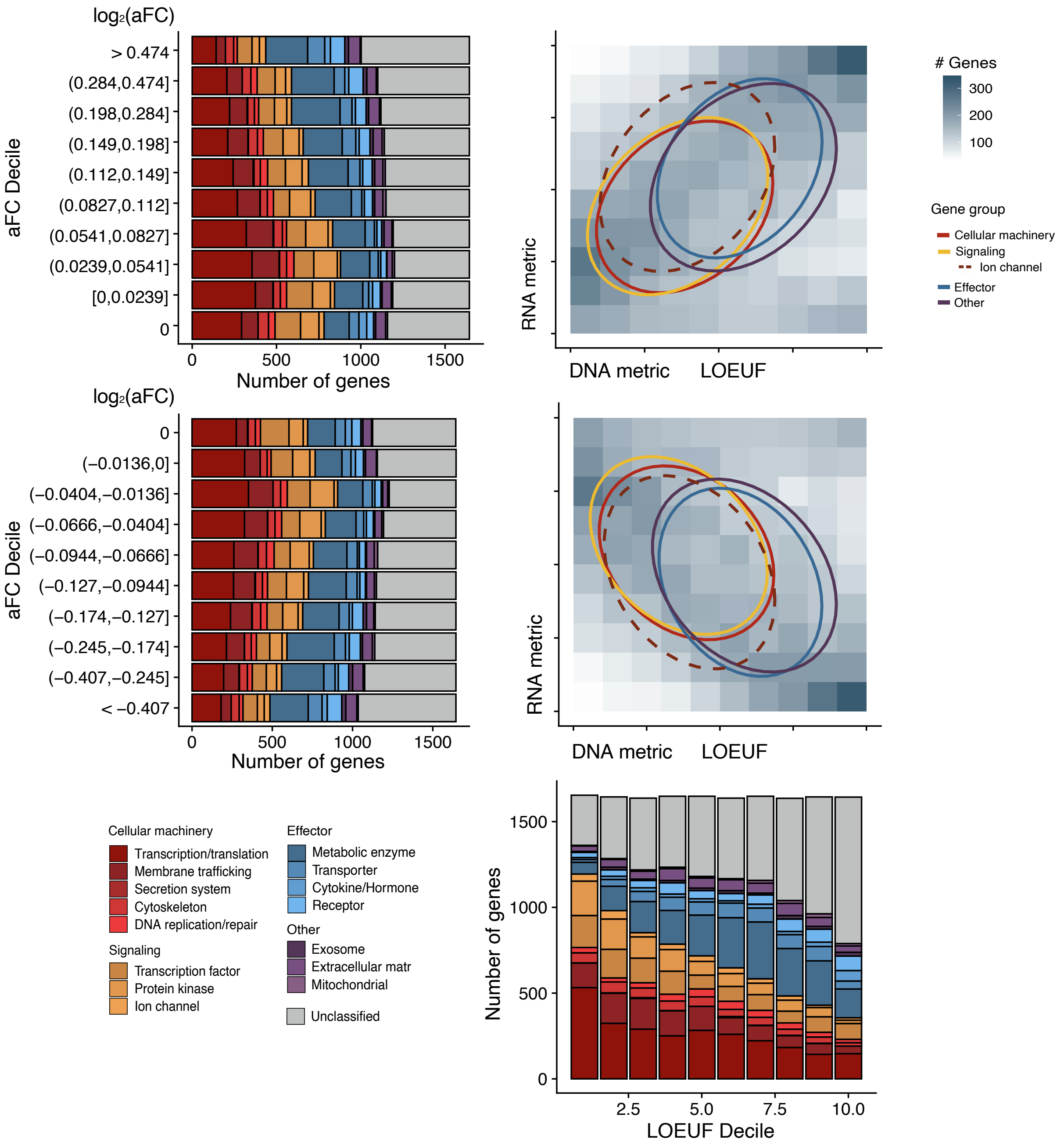

Figure S3. Dosage constraints of 16,448 autosomal protein-coding genes of different functions on DNA and RNA metrics. We show both directions of dosage change as measured by the RNA metric. Similar to Figure 2A, however the RNA metric is median aFC across tissues. Circles mark 50% of the genes around center of mass of a functional category.

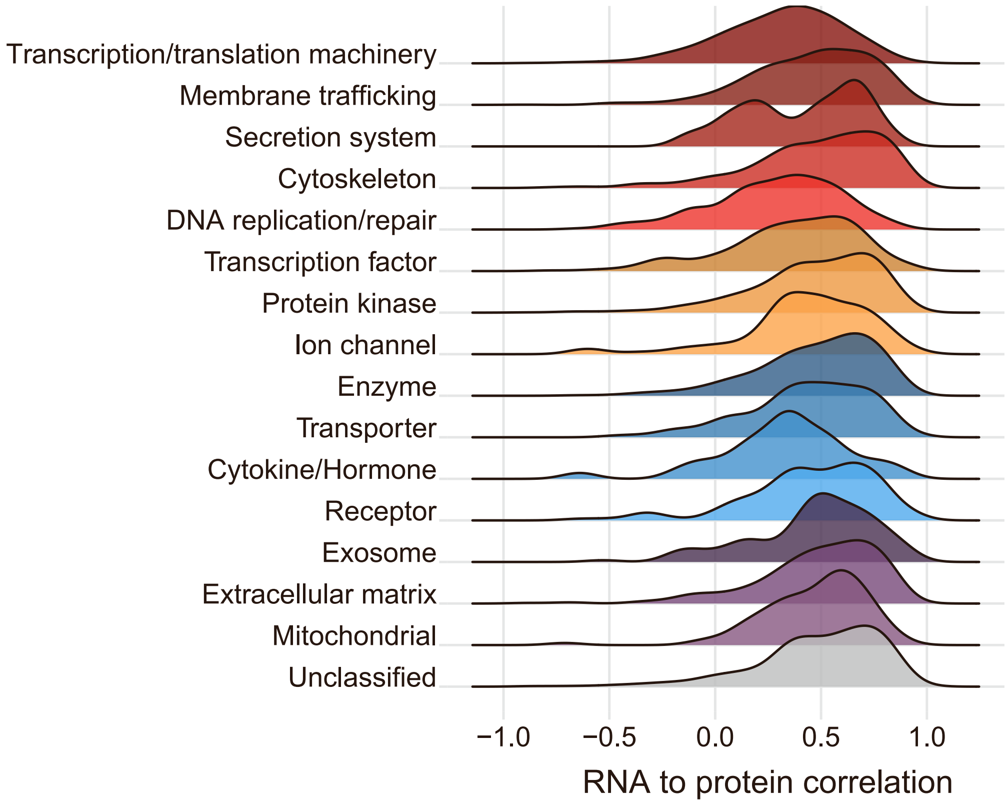

Figure S4. RNA to protein correlation. Spearman correlation (inter-individual variation) was calculated between RNA and protein levels (GTEx transcriptome and proteome data).

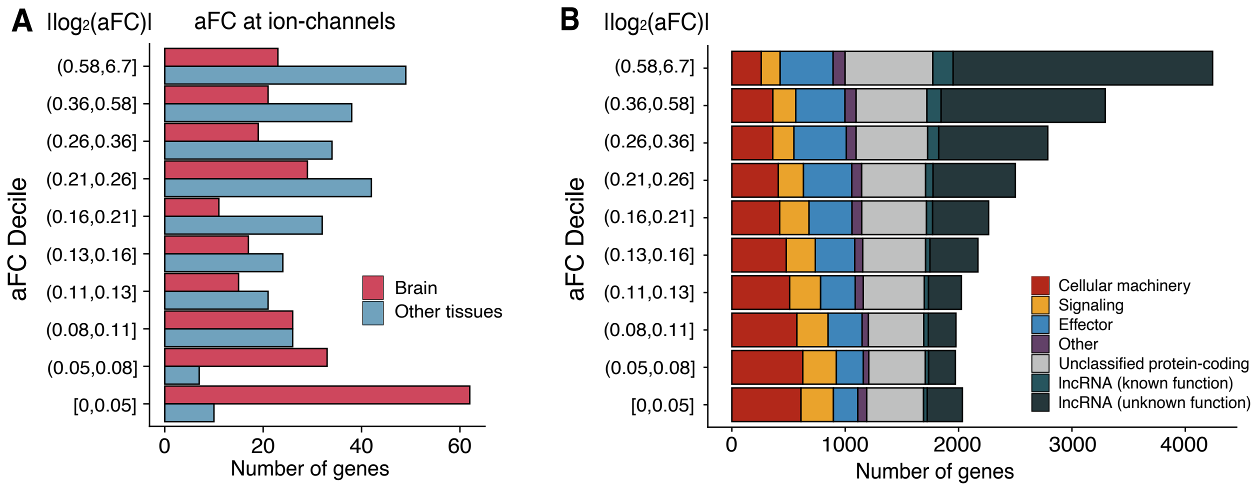

Figure S5. The RNA metric provides tissue specific information on dosage constraint of protein-coding and non-protein-coding genes. (A) Dosage constraints of ion-channels in brain and non-brain tissues (median aFC among each tissue group). (B) Comparison of dosage constraints of lncRNA with protein-coding genes. For each gene, we show median aFC across tissues.

Energy flux	Transporter	Enzyme	Physiology	Flux physical carrier & typical concentration
G_0	SGLT1		Intestine: glucose absorption	Portal vein glucose $[g_{blood}]$+
G_1	GLUT2		Liver: glucose uptake	Hepatocyte intracellular glucose = $[g_{blood}]$
L_0	FABP		Intestine: lipid absorption	Lymphatic vessel chylomicron [TG]
G_3		GYS2	Liver: glycogen turnover (glycogenesis)	$[g_{blood}]$ → glycogen
G_5		PYGL	Liver: glycogen turnover (glycogenolysis)	Glycogen → Basal blood glucose $[g_{blood}]$ ~ 4.8 ± 0.4 mmol/L
G_2	GLUT4		Muscle/adipose: insulin-response glucose uptake	After-meal blood glucose $[g_{blood}]$+
L_1		FASN	Liver: lipogenesis	VLDL [TG]
L_2	CD36		Adipose: lipid uptake	Blood total triglycerides [TG] ~ 0.9 ± 0.4 mmol/L
G_4	GLUT4	GYS1	Muscle: glycogen turnover (glycogenesis)	Intracellular glucose $[g_{cell}]$ → glycogen
G_6		PYGM	Muscle: glycogen turnover (glycogenolysis)	Glycogen→$[g_{cell}]$
L_3	GLUT4	FASN	Adipose: fatty acid synthesis	$[g_{cell}]$ → [FFA]
L_4		DGAT	Adipose: triglyceride formation	[FFA] → Lipid droplet [TG]
L_5		ATGL	Adipose: lipolysis	Lipid droplet [TG] → blood free fatty acids [FFA] 0.46 ± 0.25 mmol/L

SGLT1: sodium-glucose linked transporter 1; GLUT2: glucose transporter 2; FABP: fatty acid binding protein 1/2/6; GYS2: glycogen synthase liver isoform; PYGL: glycogen phosphorylase liver isoform; GLUT4: glucose transporter 4; FASN: fatty acid synthase; CD36: fatty acid transporter; GYS1: glycogen synthase muscle isoform; PYGM: glycogen phosphorylase muscle isoform; DGAT: diglyceride acyltransferase 1/2; ATGL: adipose triglyceride lipase; $[g_{blood}]$: blood glucose; $[g_{cell}]$: intracellular glucose; [FFA]: free fatty acids; [TG]: triglyceride

Figure S6. Energy homeostasis system. Energy flux and the metabolic enzymes and transporters those flux go through.

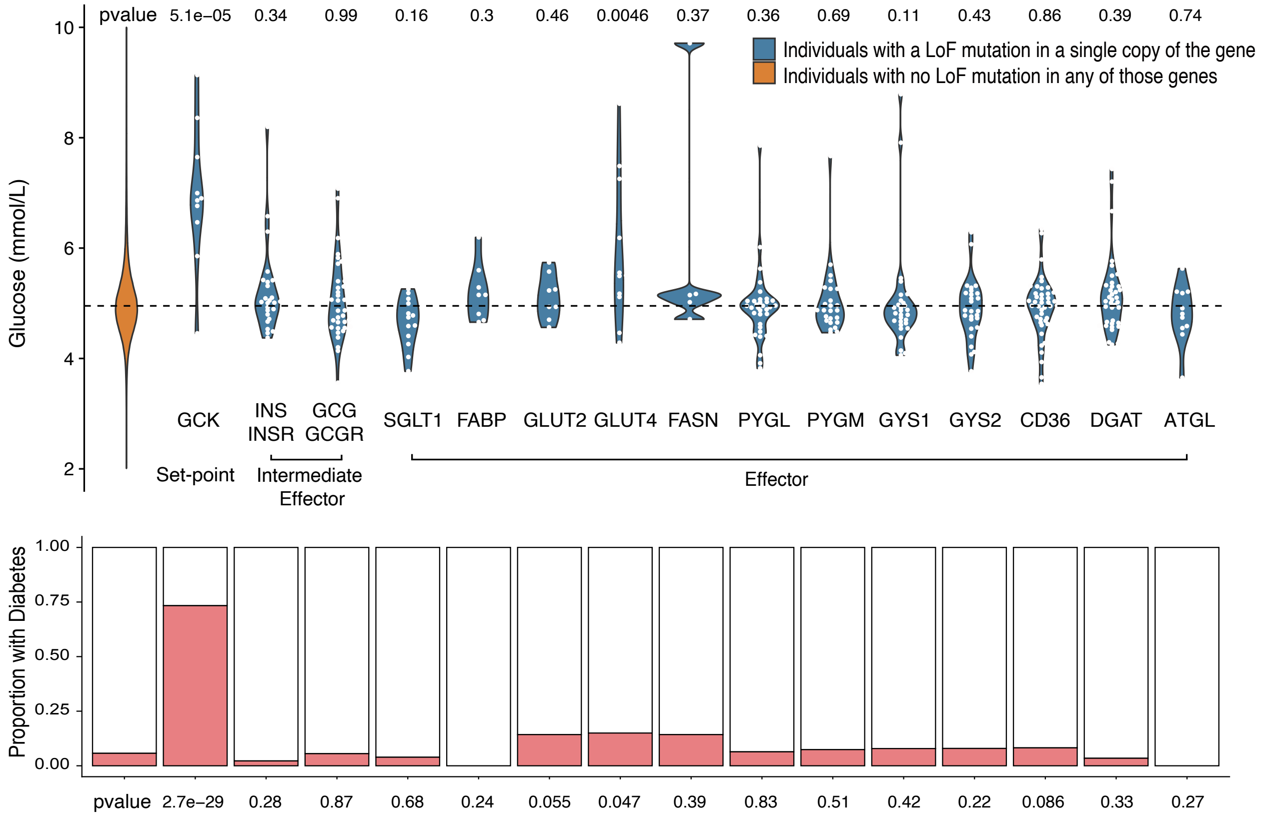

Figure S7. Glucose homeostasis in individuals with one nonfunctional copy of the gene of interest due to LoF mutations. Blood glucose levels (mmol/L) and diabetes diagnosis (see Methods) of individuals with a single LoF mutation in the gene of interest in the UK Biobank cohort.

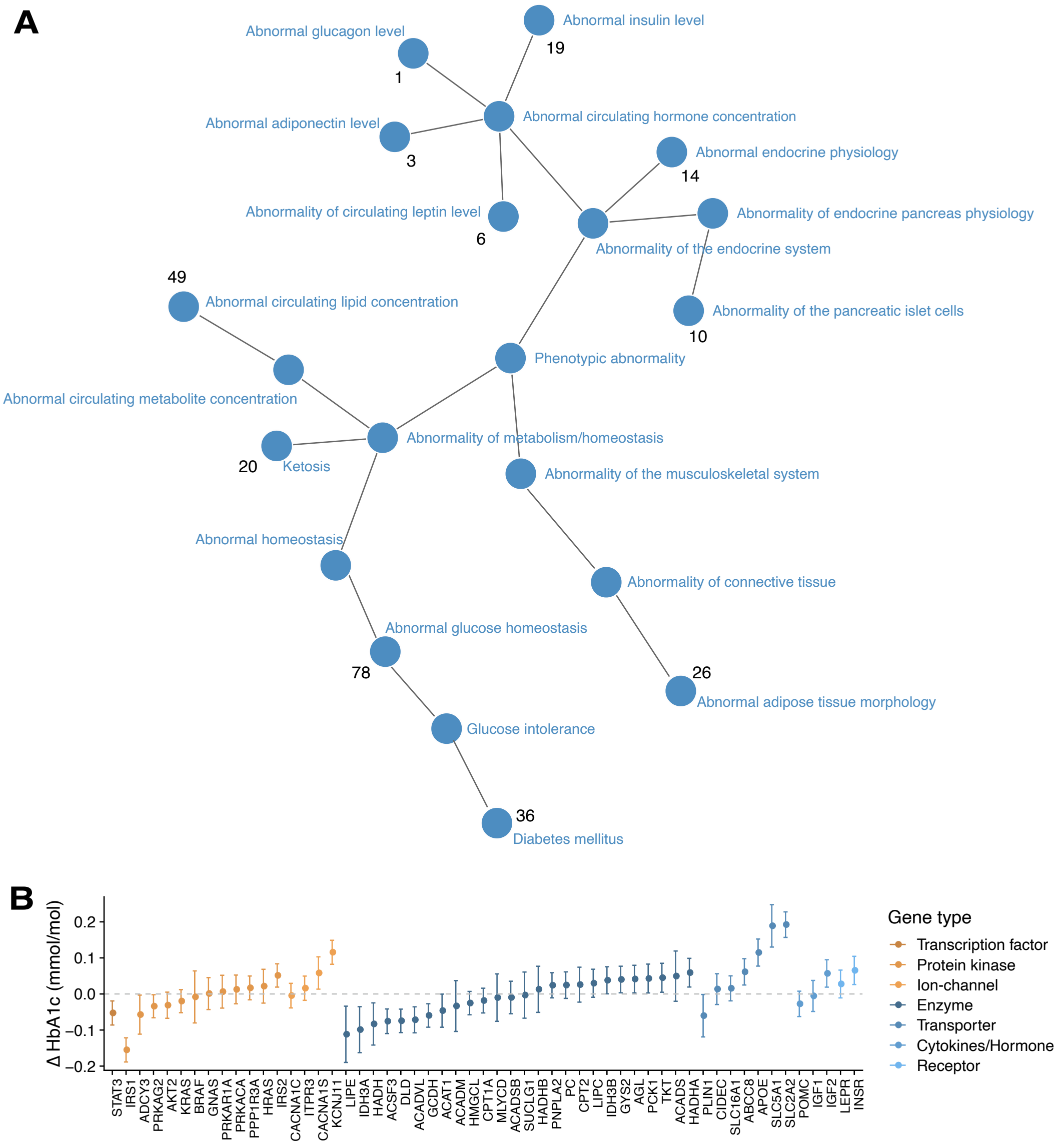

Figure S8. Phenotypes associated with variants in Mendelian disease genes and the effect of common cis-regulatory variants. (A) Major phenotypes (HPO) associated with variants in Mendelian disorder genes in the energy homeostasis system. HPO terms were organized in their hierarchical relationship. Numbers on HPO leaf nodes indicate energy homeostasis disease genes under that HPO term. (B) GWAS effect sizes of cis-regulatory variants affecting 55 Mendelian disease genes causing abnormal glucose homeostasis. 55 out of 78 genes under HPO term abnormal glucose homeostasis have abs(aFC) > 0 in major energy homeostasis tissues (intestine, stomach, liver, pancreas, muscle, adipose tissues and hypothalamus). GWAS effects (allelic change induced by a variant to glycated hemoglobin, HbA1c) in UK Biobank were evaluated among maximum aFC eQTL variants at those genes. Sign of effect indicates whether expression change increases or decreases phenotype.

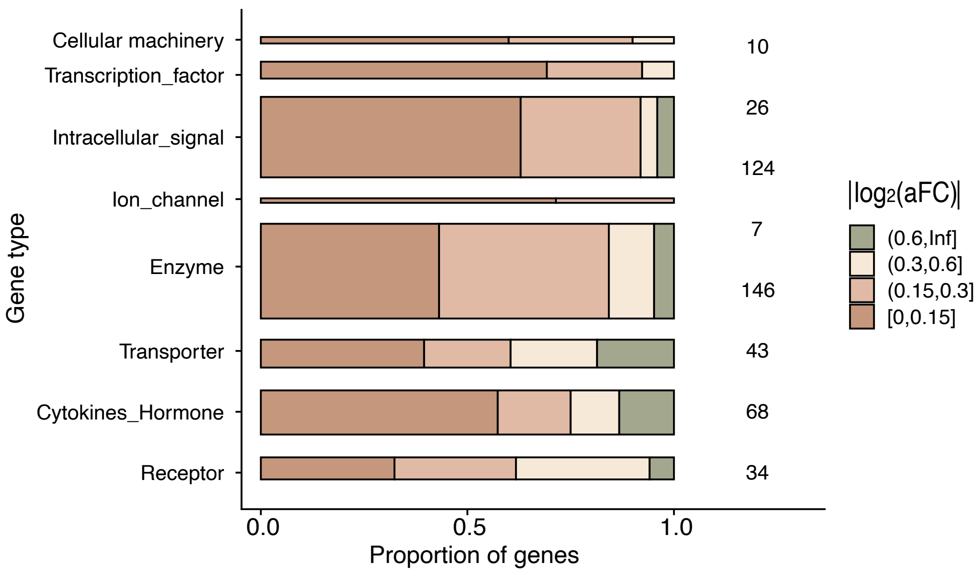

Figure S9. The RNA metric of genes in energy homeostasis system. eQTL effect sizes (aFC) of genes in each functional category (similar to Figure 5B, however showing median aFC among major organs of energy homeostasis of intestine, stomach, liver, pancreas, muscle, adipose tissues and hypothalamus).

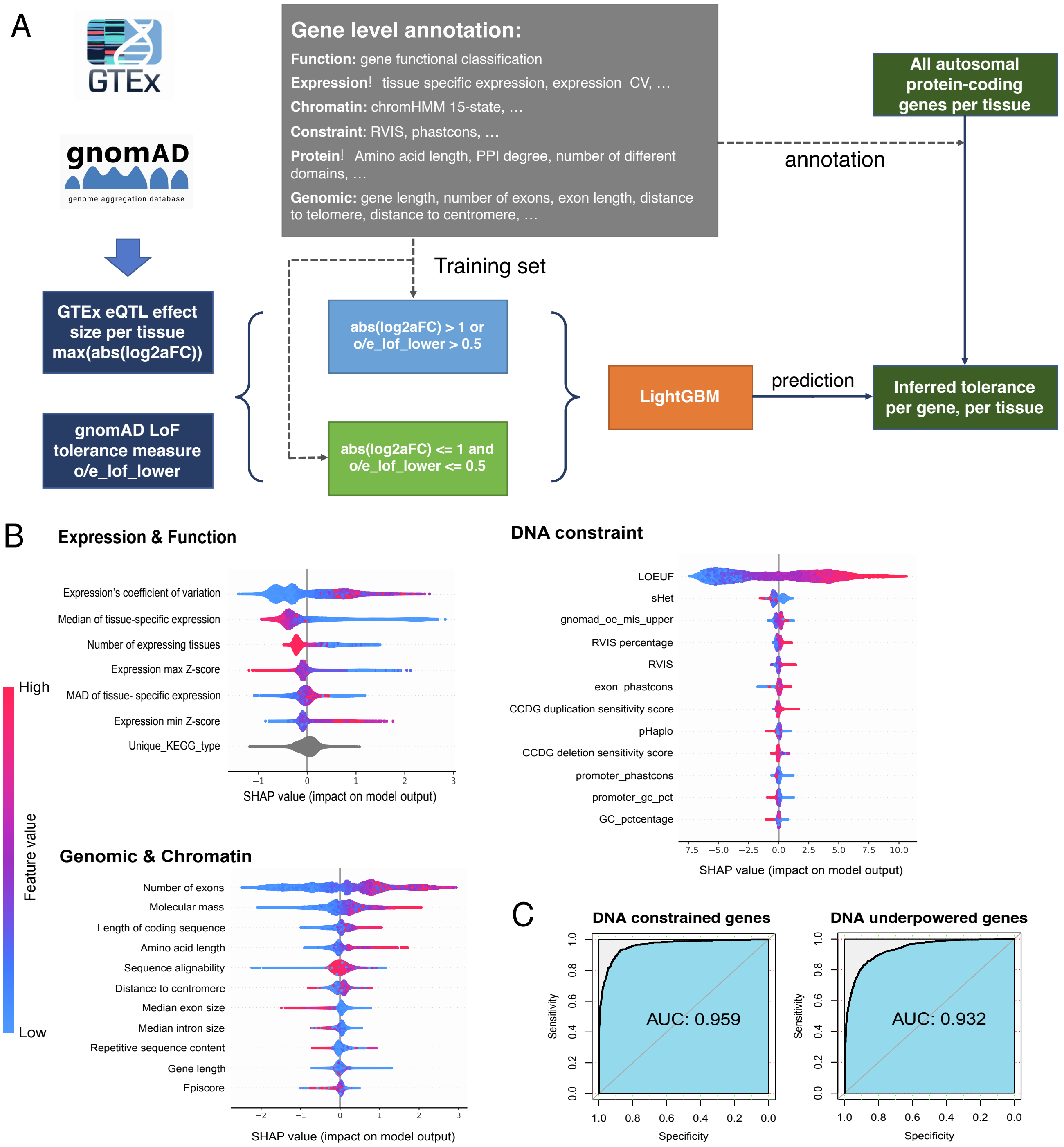

Figure S10. (A) Machine learning model to integrate the RNA and DNA dosage constraint metrics. (B) Informative features for predicting dosage constraints. Importance of features (Table S3) measured by SHAP values. (C) Predictive power of the machine learning model on held-out instances. To assess power of the model to predict tissue specific constraint beyond DNA metric constraints, AUC was assessed on DNA metric constrained genes (to predict tissue specificity) and DNA metric underpowered genes as specified in Figure 6A.

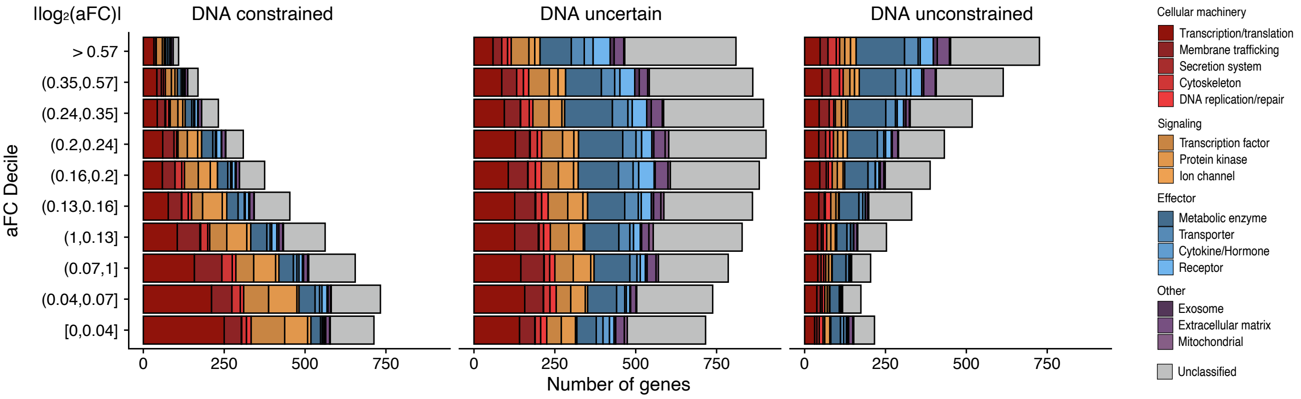

Figure S11. The RNA metric complements the DNA metric. Genes on the X axis (DNA metric) are ranked by their depletion of LoF (observed/expected, estimation lower bound). The left bin is solely composed with genes o/e upper bound < 0.5 (DNA metric high confidence constrained), while the right bin is solely with those with o/e lower bound > 0.5 (DNA metric high confidence unconstrained). The middle bin is composed of genes where the DNA metric is ambiguous (o/e lower bound < 0.5 & upper bound > 0.5). The Y axis is the RNA metric (aFC). For each gene, median aFC across tissues is plotted.

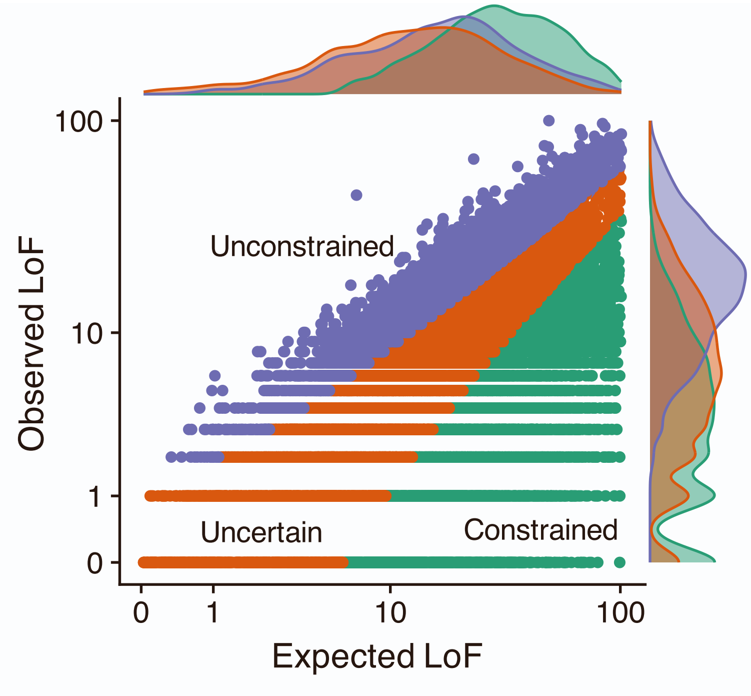

Figure S12. Grouping of genes on the DNA metric. The DNA metric (observed/expected LoF) confidence interval is influenced by gene length. Shorter genes (small expected LoF count) will have wider confidence interval thus more ambiguous to be assigned as either constrained or tolerant.

Supplementary Tables

Table S1. Functional classification of genes.

Table S2. List of modules and genes of energy homeostasis system. KEGG pathways and HPO phenotype hierarchy associated with energy homeostasis.

Table S3. Features used in the machine learning model.

Table S4. aFC of genes across 49 tissues.

Table S5. RNA integrated score (MoDs) of genes across 49 tissues.
